# Case Report: Deep Brain Stimulation of the Nucleus Basalis of Meynert for Advanced Alzheimer's Disease

**DOI:** 10.3389/fnhum.2021.645584

**Published:** 2021-05-26

**Authors:** Wei Zhang, Wei Liu, Bhavana Patel, Yingchuan Chen, Kailiang Wang, Anchao Yang, Fangang Meng, Aparna Wagle Shukla, Shanshan Cen, John Yu, Adolfo Ramirez-Zamora, Jianguo Zhang

**Affiliations:** ^1^Center for Cognitive Neurology, Department of Neurology, Beijing Tiantan Hospital, Capital Medical University, Beijing, China; ^2^Department of Neurosurgery, Beijing Tiantan Hospital, Capital Medical University, Beijing, China; ^3^Department of Neurology, Norman Fixel Institute for Neurological Diseases, University of Florida, Gainesville, FL, United States; ^4^Department of Functional Neurosurgery, Beijing Neurosurgical Institute, Capital Medical University, Beijing, China; ^5^Beijing Key Laboratory of Neurostimulation, Beijing, China

**Keywords:** deep brain stimulation, nucleus basalis of Meynert, Alzheimer's disease, thermoregulatory disturbance, Mini-Mental State Examination

## Abstract

Patients with advanced Alzheimer's disease (AD) experience cognitive impairment and physical disabilities in daily life. Currently, there are no treatments available to slow down the course of the disease, and limited treatments exist only to treat symptoms. However, deep brain stimulation of the nucleus basalis of Meynert (NBM-DBS) has been reported to improve cognitive function in individuals with AD. Here, we report the effects of NBM-DBS on cognitive function in a subject with severe AD. An 80-year-old male with severe AD (Clinical Dementia Rating scale: 3.0 points) underwent surgery for bilateral NBM-DBS electrode placement. After 10 weeks of stimulation, Mini-Mental State Examination (MMSE) assessment improved from a score of 5 to 9 points, and assessment using the Alzheimer's Disease Assessment Scale–Cognitive Subscale (ADAS-cog) showed a marked reduction in total score from 43 to 33 points, suggesting cognitive benefits from NBM-DBS. The patient's postoperative course was complicated by a subdural effusion that occurred several days after surgery, with complete recovery. Interestingly, the subject also displayed abnormal thermoregulation with stimulation initiation and stimulation parameter modifications. NBM-DBS may serve as a potential therapy for severe AD patients.

**Clinical Trial Registration:** ChiCTR1900022324.

**Graphical Abstract:**
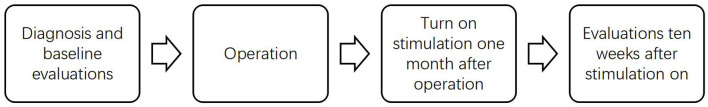
Interventions timeline.

## Introduction

Alzheimer's disease (AD), a neurodegenerative disease, is the most common type of dementia, affecting ~24 million people worldwide; a number expected to quadruple by 2050 (Lozano et al., 2016; Association As, 2018). Current therapeutic approaches are very limited and do not provide significant benefits for AD patients.

The degeneration of cholinergic neurons is one of the most important pathological features of AD, and increasing brain acetylcholine (Ach) with acetylcholinesterase inhibitors can improve overall abilities and cognitive function in AD patients. The nucleus basalis of Meynert (NBM), located in the basal forebrain, is the primary source for cholinergic innervation to the neocortex and amygdala (Lee et al., 2019). The human NBM can be subdivided into six regions: anteromedial (Ch4am), anterolateral (Ch4al), anterointermediate (Ch4ai), intermediodorsal (Ch4id), intermedioventral (Ch4iv), and a posterior (Ch4p) region (Liu et al., 2015). Increasingly, NBM deep brain stimulation (NBM-DBS) has been used to treat patients with AD. It was first performed in patients with AD during the 1980s with positive effects on ipsilateral cerebral metabolism using positron emission tomography (PET) assessment (Turnbull et al., 1985). Recently, Kuhn et al. (2015) reported the outcomes of six patients with AD who were treated with bilateral low-frequency DBS that targeted Ch4id, Ch4iv, or Ch4q NBM areas. In their report, four patients were considered “responders” with either improvements or no changes in cognition during 12-month follow-ups; no severe or unexpected side effects were observed (Kuhn et al., 2015). They suggested that DBS might slightly improve or stabilize symptoms in some AD patients. However, they argued that beneficial effects of NBM-DBS were more apparent in younger and lesser-affected patients, and most DBS studies have only focused on mild to moderate disease in AD patients (Kuhn et al., 2015; Baldermann et al., 2018). In severely affected AD patients, who urgently need cognitive improvement, the effects of DBS remain unknown. Here, we report the outcome of an AD patient with severe disease treated with bilateral NBM-DBS.

## Case Description

An 80-year-old right-handed male presented at our hospital with a 4-year decline in episodic memory and major cognitive deterioration for half a year. In the 4 years prior to presentation, the patient had gradually begun to show a decline in memory, hesitant speech, and impaired organizational skills. He was first diagnosed with AD at another hospital and treated with donepezil (5 mg/day). His symptoms remained tolerable until 3 years later, when he could no longer perform independent daily living activities. Six months prior to presentation, he could not recognize his family members and common objects. At that time, he was being treated with donepezil (5 mg/day), rivastigmine (3 mg/day), and memantine (10 mg/day).

The patient had a 40-year history of hypertension that was controlled well with levamlodipine besylate tablets (2.5 mg/day) and fosinopril (10 mg/day). There was no history of psychological disease or any family-related history. A clinical examination showed that he could not answer questions correctly or fluently.

**Figure 1:**
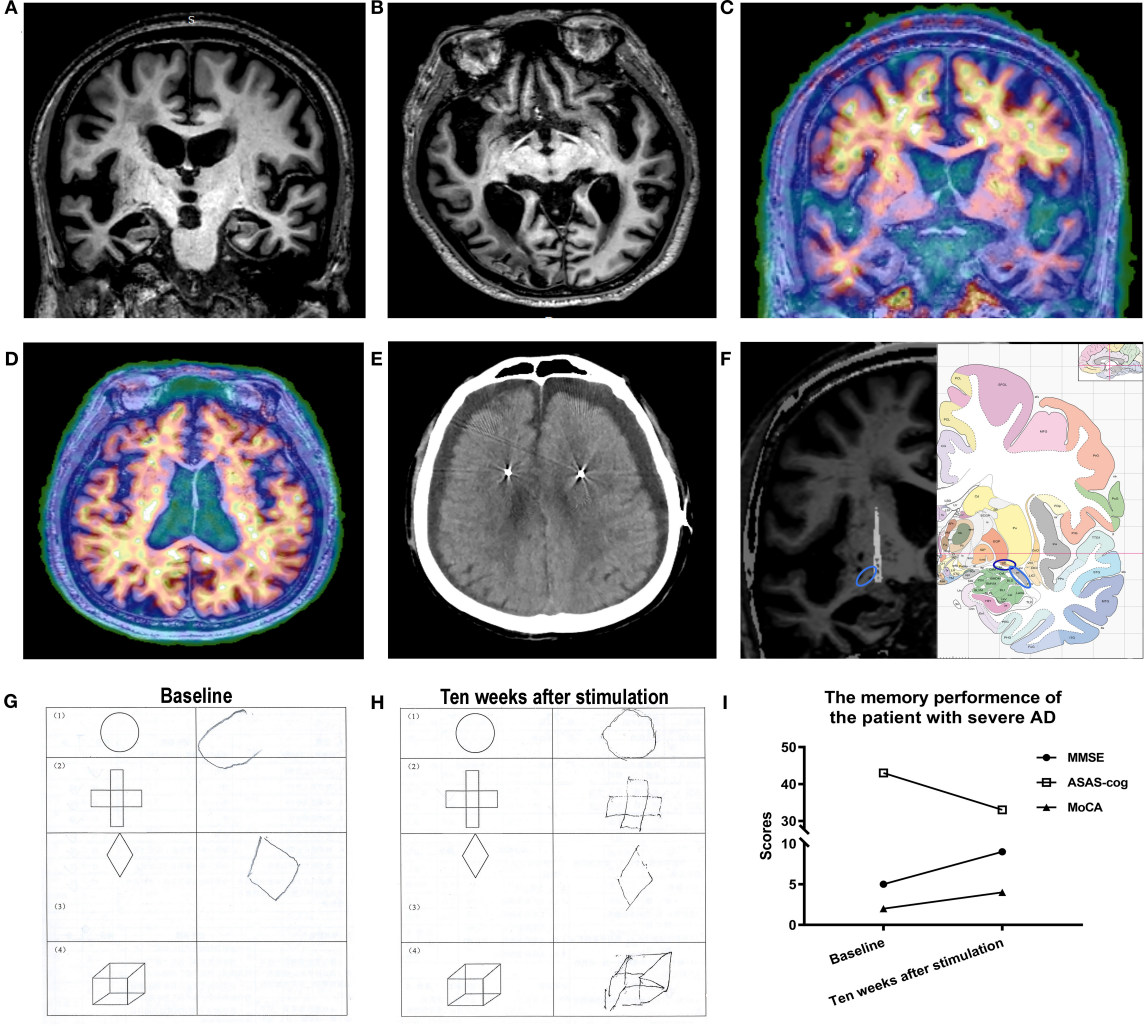
**(A,B)** A preoperative MRI revealed global atrophy, especially in the hippocampi and temporal lobes. **(C,D)** PET imaging showed β amyloid widely deposited in the brain. **(E)** Bilateral subdural effusion was identified by CT 1 week after surgery. **(F)** A fused image (left) of the patient's preoperative MRI and postoperative CT showed that the lead (arrow) was accurately implanted into the nucleus basalis of Meynert (NBM) (dark blue circle = NBM; light blue circle = anterior commissure). Atlas (right) of the human brain. **(G,H)** Patient performance before and after NBM-deep brain stimulation (DBS). The patient was able to draw all geometric shapes after NBM-DBS but performed poorly before NBM-DBS. **(I)** Changes in memory performance by the patient over time.

Magnetic resonance imaging (MRI) demonstrated severe volume loss of the whole brain (Figures 1A,B), and PET showed that β amyloid (Aβ) was widely deposited throughout the whole brain (Figures 1C,D), with the dorsal cortex having more Aβ than the medial temporal lobe and indicating end-stage AD according to Braak staging (Braak and Braak, 1991).

This AD assessment was made at Beijing Tiantan Hospital, Capital Medical University, China. As no effective AD treatments exist, his family wanted to try DBS treatment. In December 2018, the patient and his family members were fully informed about the risks and possible ineffectiveness of DBS treatment before they agreed and signed the consent form for this NBM-DBS study, which was approved by the ethics committee of Beijing Tiantan Hospital (No.KY 2018-051-02).

Preoperative clinical examinations showed no contraindications for the surgery. As the temporal brain regions (with projections from Ch4p) showed less Aβ deposition in this patient, and Ch4p area was easily targeted given its location relative to the anterior commissure, planning called for placement of the electrode leads to target the Ch4p area of the NBM based on MRI scans and a human brain atlas (Mai et al., 2015). [Detailed target planning method, see Liu and Yu (2020)].

For DBS, the patient underwent bilateral lead placement using a rechargeable implantable pulse generator (L301, Beijing PINS Medical Co. Ltd., Beijing, China; lead diameter, 1.3 mm; electrode length, 1.5 mm; electrode spacing, 0.5 mm; distance from distal tip, 0.5 mm). Briefly, the Leksell stereotactic system was used on the patient, and MRI scans were performed for targeting purposes. Left and right burr holes were drilled in the skull according to planning-derived coordinates, and the arc and ring settings (Table 1).

**Table 1:** Target coordinates and stimulation parameters.

	**Target coordinates** **(x, y, z) (mm)**	**Contact**	**Stimulating setting**
Right hemisphere	20.3, −3.6, −6.2	C+ 1–	20 Hz 90 μs 1.5 V
Left hemisphere	−23.5, −6.2, −6.5	C+ 5–	20 Hz 90 μs 1.5 V

Postoperative computerized tomography (CT) demonstrated no evidence of acute territorial infarcts, focal mass lesions, or midline shifts and confirmed the locations of the electrode placements. One week after surgery, the patient had altered consciousness, impaired alertness, and loss of appetite. Reexamination of the patient using CT revealed subdural effusion (Figure 1E). Patient symptoms were treated conservatively and resolved over the following weeks.

DBS stimulation began 1 month after surgery using a monopolar protocol. Based on previous studies (Turnbull et al., 1985; Dürschmid et al., 2017) and the clinical imaging results, we used monopolar stimulation from the bottom contact (1-, 5-, product model 106R, PINS, China) for this patient. The stimulation frequency and pulse width were maintained at 20 Hz and 90 μs, respectively, and the starting voltage of 1 V was gradually increased to 1.5 V.

Unexpectedly, the patient's body temperature rose from 36.5 to 37.7°C, and he experienced mood fluctuations, loss of appetite, and abnormal eating behaviors, which all resolved back to normal 2 days later (36.2–36.4°C). As these symptoms were reduced 3 days after turning on the implanted pulse generator, we decided to increase the stimulation voltage, and the patient's temperature rose to 37.7°C again and the other symptoms described above reappeared. As before, the patient's temperature returned to normal by 3 days without any special treatment. As a result, to avoid temperature fluctuations, the stimulation parameters of 1.5 V, 20 Hz, and 90 μs (1-, case+) were set and maintained for the patient.

A postoperative CT scan was fused with a preoperative MRI to localize the lead positions and demonstrated that they were accurately implanted into the NBM (Figure 1F). A previous study reported that the NBM extended 25 mm lateral to the midline, 13 mm ventral to the superior edge of the anterior commissure (AC) at the midline, and 3 mm anterior to and 9 mm posterior to the middle of the AC (Teipel et al., 2005). In this study, the coordinates of the left planning target were 23.5 mm lateral to the midline and 6.5 mm ventral and 6.2 mm posterior to the AC, and the coordinates of the right planning target were 20.3 mm lateral to the midline and 6.2 mm ventral and 3.6 mm posterior to the AC, which were all consistent with the previous study (Teipel et al., 2005).

At baseline, this patient had severe dementia indicated by a Clinical Dementia Rating of 3.0 points. After 10 weeks of NBM stimulation, cognitive evaluations were also conducted. Compared to baseline assessments, the patient displayed an improvement in the Alzheimer's Disease Assessment Scale–Cognitive Subscale (ADAS-cog), from 43 to 33 points, with changes in word recall (1-point reduction), naming objects and fingers (1-point reduction), commands (2-point reduction), constructional praxis (2-point reduction), ideational praxis (2-point reduction), word recognition task (3-point reduction), and remembering test instructions (1-point increase) (Figure 1I), suggesting a significant improvement with NBM-DBS in the short term.

In addition, Mini-Mental State Examination (MMSE) scoring improved from 5 to 9 points with an improvement in location ability, and Montreal Cognitive Assessment scoring increased from a score of 2 to a score of 4 with improvements in both attention and orientation. Notably, the patient's executive functions (structure identification and imitation) also improved (Figures 1G,H), and he could remember the names of his children during NBM-DBS—something he could not do before DBS. His wife also reported such improvements; however, she did not feel substantial changes during daily life; a caregiver was still needed.

## Discussion

In recent years, NBM-DBS has been investigated as a potential treatment for AD, but only a few cases have been reported (Kuhn et al., 2015; Baldermann et al., 2018), so additional safety and efficacy studies for the procedure in AD are needed.

Previous NBM-DBS studies have examined individuals with mild to moderate AD (Kuhn et al., 2015; Dürschmid et al., 2017), so the effects of such stimulation in patients with severe AD have not been consistently identified. We have demonstrated that NBM-DBS was effective in this case of advanced-stage AD, and despite initial perioperative complications, we observed a marked improvement in the patient's cognitive function in the short term (a 23.3% decrease in ADAS-cog scoring) without any changes to the patient's medications.

In previous studies, the targeting regions for the electrodes were not specified (Kuhn et al., 2015; Baldermann et al., 2018), so the results could not be differentiated according to NBM areas, and potentially effective regions were unclear. In this study, the Ch4p region was targeted. Ch4p cholinergic neurons project to the temporal poles and superior temporal regions, so any improvements should be correlated with temporal-region functions and closely related to memory. Dürschmid et al. (2017) suggested that NBM-DBS has a positive impact on sensory gating into memory, with a beneficial effect on the recognition of familiar stimuli. Interestingly, the patient's postoperative improvements, including word recall, word recognition tasks, and the naming of objects, were all related to memory functions.

In this patient, PET imaging showed fewer Aβ deposits in temporal regions (Figure 1C), which may have contributed to his improvements. However, according to Braak staging (Braak and Braak, 1991), most severe AD patients were similar to this patient, with fewer Aβ deposits in temporal brain regions, suggesting that similar improvements should also be possible in other advanced-stage patients. Strangely, the dorsal cortex white matter in this patient seemed to have more Aβ than the gray matter. We interpret this finding to be a possible manifestation of end-stage disease. As the distribution of amyloid deposits in the white matter have been correlated with blood vessels (Iwamoto et al., 1997; Charidimou et al., 2015), there may be more blood vessel damage with Aβ deposits in end-stage disease, and severe atrophy of the brain could lead to more perivascular spaces with more Aβ deposits. In addition, soluble Aβ levels may simply be high in white matter in end-stage AD (Collins-Praino et al., 2014).

Previous studies have demonstrated that the NBM degenerates (thus decreasing Ach release) in AD patients, but stimulation of the NBM may increase Ach release in the cortex (Kuhn et al., 2015; Gratwicke et al., 2018). In the past, NBM-DBS was not recommended for severe AD patients because of the few surviving neurons in the NBM. However, the current study suggests that NBM-DBS might increase Ach release and improve cognitive function, even in patients with severely compromised NBMs. The Ch4p group of neurons is considered to suffer the most severe loss of cholinergic cells in AD patients, but in this patient, the results demonstrate that DBS stimulation of the Ch4p regions was still efficacious, although we cannot exclude the possibility of anatomical individuality for this patient.

This patient suffered from bilateral, perioperative subdural effusions after the lead implantations. Possibly related to advanced age and severe brain atrophy, overall subdural effusions have been reported to be rare and to mainly occur as a result of subdural manipulations (Saitoh et al., 2001). Although postoperative subdural effusions did occur, the patient did recover from them.

A thermoregulatory disorder occurred in this patient during the initiation of stimulation, and with adjustments of the stimulation parameters. Pathological markers for AD have been found in the hypothalamus, which could account for the thermoregulatory dysfunction (Van de Nes et al., 2006), and stimulation of the NBM may have made it worse. With severe brain atrophy, the distance between the NBM and the hypothalamus is reduced and increases the likelihood of aberrant stimulation effects in the hypothalamus. Another possible explanation is that an increase in Ach, due to NBM-DBS stimulation, may have activated cold sensors, leading to a change in body temperature. Based on the temperature data for this patient, we postulate that the hypothalamic thermoregulatory function returned to normal several days after neurostimulation initiation.

### Limitations

As Xu and Ponce (2018) argued, the choice of stimulation parameters for NBM-DBS has been somewhat arbitrarily derived from animal models; varying stimulation settings may significantly impact the biological effects of therapy. We chose stimulation parameters just according to the study by Kuhn et al. (2015). These might not be the most effective parameters. Variable parameters should be tested in future studies. In this study, only one AD patient with severe disease was treated with NBM-DBS, so generalized conclusions cannot be drawn. Besides, it is a pity that postoperative PET, which is a powerful tool to show metabolic changes and to elucidate the mechanism of cognitive changes after NBM-DBS, was not implemented on this patient. Data from additional patients are being collected to confirm these results, and future studies will explore the detailed mechanisms underlying the effect of NBM-DBS in severe-stage AD patients.

## Summary

We report a potential therapeutic option for AD patients with severe disease. If transient thermoregulatory disturbances and subdural effusions occur, they should be managed carefully.

## Data Availability Statement

The original contributions presented in the study are included in the article/supplementary material, further inquiries can be directed to the corresponding authors.

## Ethics Statement

The studies involving human participants were reviewed and approved by Ethics Committee of Beijing Tiantan Hospital (No.KY 2018-051-02). Written informed consent to participate in this study was provided by the participants' legal guardian/next of kin. Written informed consent was obtained from the individual(s), and minor(s)' legal guardian/next of kin, for the publication of any potentially identifiable images or data included in this article.

## Author Contributions

JZ designed the research. WZ contributed to the diagnosis and evaluation of the AD patient and revised the manuscript. WL, KW, AY, FM, and JZ performed the operation. WL, BP, AWS, YC, SC, JY, and AR-Z wrote and revised the manuscript. All authors contributed to the article and approved the submitted version.

## Conflict of Interest

The authors declare that the research was conducted in the absence of any commercial or financial relationships that could be construed as a potential conflict of interest.
